# Stabilization of Metastable Indomethacin α in Cellulose Nanocrystal Aerogel Scaffolds

**DOI:** 10.3390/pharmaceutics13040441

**Published:** 2021-03-24

**Authors:** Manali Banerjee, Blair Brettmann

**Affiliations:** 1 School of Materials Science and Engineering, Georgia Institute of Technology, Atlanta, GA 30332, USA; bmanalib@gatech.edu; 2 School of Chemical and Biomolecular Engineering, Georgia Institute of Technology, Atlanta, GA 30332, USA

**Keywords:** crystallization, indomethacin, cellulose nanocrystal, aerogel, polymorphism, Raman spectroscopy, metastability, polymorph stabilization

## Abstract

Indomethacin (IM) is a small molecule active pharmaceutical ingredient (API) that exhibits polymorphism with the γ-form being the most thermodynamically stable form of the drug. The α-form is metastable, but it exhibits higher solubility, making it a more attractive form for drug delivery. As with other metastable polymorphs, α-IM undergoes interconversion to the stable form when subjected to certain stimuli, such as solvent, heat, pH, or exposure to seed crystals of the stable form. In this study, IM was crystallized into cellulose nanocrystal aerogel scaffolds as a mixture of the two polymorphic forms, α-IM and γ-IM. Differential scanning calorimetry (DSC) and Raman spectroscopy were used to quantitatively determine the amount of each form. Our investigation found that the metastable α-IM could be stabilized within the aerogel without phase transformation, even in the presence of external stimuli, including heat and γ-IM seed crystals. Because interconversion is often a concern during production of metastable forms of APIs, this approach has important implications in being able to produce and stabilize metastable drug forms. While IM was used as a model drug in this study, this approach could be expanded to additional drugs and provide access to other metastable API forms.

## 1. Introduction

Active pharmaceutical ingredients (APIs) often exhibit polymorphism, the phenomenon of packing into different crystal structures while having the same chemical composition. Each polymorph has unique physical and chemical properties, including shape, purity, and free energy [1,2,3], which can alter their behavior in terms of stability and solubility [4,5,6]. Indomethacin (IM) is a nonsteroidal anti-inflammatory drug that is often used as a model compound for studies that are related to polymorphism [7,8,9]. IM is poorly water-soluble and it exists in at least five polymorphic forms, with the γ form being the most thermodynamically stable and the α form as the most commonly occurring metastable form [8,10]. Table 1 shows the crystal data and physical properties of the two key polymorphs (α-IM and γ-IM) discussed in this paper. Previous studies have reported that α-IM has higher solubility than the γ form, owing to its metastable nature as well as the conformation of the carboxyl groups within the polymorph structure [11,12,13].

Unfortunately, accessing and stabilizing metastable polymorphs remains a major challenge in the field of API production and formulation [6,17]. These polymorphs often require very specific conditions to crystallize, including control over solvent, temperature, pH, and level of supersaturation [1,18]. Recently, a number of studies have reported ways for obtaining metastable forms of certain APIs using surface templating [19,20,21], solid dispersions [16,18], or confinement [22,23,24]. However, studies into stabilizing the metastable forms are limited. Metastable polymorphs are unstable and interconvert into the stable API when subjected to certain stimuli, such as moisture, solvent, heat, pH, or even time [25,26]. Research into polymorph interconversion and ways to stabilize polymorphs against interconversion remains an emerging field, with a few studies looking at nanoconfinement [27] or additives [20,26,28,29] to stabilize the metastable forms. The interconversion of metastable drugs often leads to limitations with analysis, resulting in insufficient data for certain API forms [30,31]. Solvent mediated transformations prevent the accurate measurements of solubility for certain API polymorphs [32]. For some drug forms, including the metastable form III of acetaminophen, solid state transformation can also occur immediately after preparation or within hours without any external stimuli, preventing thorough investigation of this form [31,33,34,35]. This limits the use of metastable forms of a drug in commercial applications, even though they may exhibit higher therapeutic activity.

The interconversion of α-IM to γ-IM, specifically, can occur due to heat, solvent, mechanical action, such as milling, and exposure to the more stable γ form [12,36,37,38]. This transformation has been reported in several previous studies and it follows Ostwald’s rule of phases with the metastable α-form converting to the more stable γ-form [9,39]. α-IM has not previously been reported to convert to other polymorphic forms. The α to γ transition has previously led to limitations in studying α-IM, especially while using thermal analysis techniques [7]. Interconversion has also caused issues with using α-IM in hot melt extrusion, an emerging technique for downstream API processing [37]. It has also been suggested that the presence of small amounts of γ-IM can result in the transformation of the α form [12], indicating that any impurities or γ seeds during production could lead to interconversion and lower the solubility of the final drug products. Previously, this seed effect has only been studied in the presence of a solvent and the need for studying this interconversion in the solid state exists. Additionally, as far as we could find, no groups have investigated the stabilization of the solid α-IM form in the presence of γ seed crystals.

Our approach to stabilizing metastable polymorphs uses cellulose, which is an abundant biopolymer, as an excipient. Cellulose and its derivatives have been used in the pharmaceutical industry for many decades, serving as excipients, fillers, matrices, and drug delivery platforms for a variety of oral dosage forms [40,41,42]. More recently, the nanoscale form, cellulose nanocrystals (CNCs), have been studied as systems for controlled drug release and stabilizing particles [42,43,44]. Nanocellulose aerogels are an exciting new field of light-weight materials that have high surface area and open porosity that can be loaded with active ingredients [45,46]. These materials have been successful in the formation and stabilization of micro and nanoscale particles, including polymer particles [47], silver nanoparticles [48], and APIs [49,50,51,52]. Nanocellulose aerogels are self-standing scaffolds that have a large number of functional groups that are capable of electrostatic and hydrogen bonding interactions with the APIs and are composed of pores to provide a confined environment [49,52]. Hydrogen bonding with nanocellulose has been used to direct the crystallization of specific polymorphs of APIs, including carbamazepine, acetaminophen, and fenofibrate, showing promise as heterogeneous surfaces for controlling crystallization [53]. However, using CNCs to stabilize metastable drug forms has not previously been reported.

In this work, we use CNC aerogels as scaffolds to crystallize and stabilize the metastable α-form of indomethacin. While α-IM is the more soluble form, it has the tendency to undergo interconversion to the more stable γ-form, lowering the bioavailability of the drug when administered to the body. By directly crystallizing within CNC aerogels, α-IM crystals are obtained and stabilized within a scaffold and phase transformation is delayed. Thorough Raman spectroscopy studies show that the metastable α-IM is stable in the CNC aerogels against seeding effects from γ-IM crystals, which are otherwise sufficient in inducing interconversion. 

## 2. Materials and Methods

Indomethacin (γ-form) and acetone were purchased from Acros Organics (Fair Lawn, NJ, USA). Ethanol was purchased from Decon Labs (King of Prussia, PA, USA). The as-received API was confirmed to be γ-form using differential scanning calorimetry (DSC) and Raman spectroscopy, as shown in the results section of this paper. Cellulose nanocrystals were obtained from Celluforce as a spray dried powder (Lot # 2015-009). More information on the physical properties of the CNCs can be found in our previous publication [54].

Indomethacin α-form was prepared, as described in previous studies [7,9], by first dissolving γ-IM in ethanol, followed by adding deionized water to precipitate the α-IM form. The obtained α-IM crystals were vacuum filtered out of the solution and then dried under vacuum at room temperature and will be denoted α-IM in the paper.

CNCs were washed with acetone in order to purify and improve the reproducibility of the materials, as described in our previous work [54]. Briefly, CNC powder was suspended in acetone and stirred for 10 min. The dispersion was then centrifuged at 10,000 RPM for 10 min. using an Eppendorf centrifuge. The supernatant was removed, and the wash-centrifuge cycle was repeated twice more. The purified CNCs were left to dry in air overnight.

CNC aerogels were made by freeze drying 1% CNC suspensions. First, 1 wt% CNC suspensions in water were probe sonicated to fully disperse the material. 5 mL samples of the suspension were then placed into a silicone tray and the tray was put into a −80 °C freezer overnight. The frozen material was then freeze dried using a FreeZone 2.5 L freeze dryer. The aerogels were hexagonal prisms with an average density of 0.0124 mg/mm^3^ based on their size and mass. SI Figure S1 includes images of the aerogels.

Crystallization within CNC aerogels was performed by drop casting IM solutions into CNC aerogels. First, a 30 mg/mL solution of γ-IM in ethanol was heated to 50 °C to fully dissolve the material. 1 mL of this solution was cast into CNC aerogels that were held within the silicone tray to prevent liquid from leaking out. The aerogels containing the IM solution were then placed in a fridge at 10 °C to induce crystallization. Finally, the aerogels were stored under vacuum overnight to remove any excess solvent. These samples are called IM-aerogel in the paper.

α-IM samples were tested for stability in two ways. One was stability against interconversion in the presence of γ-IM seeds and the second was a stability under heated conditions. In order to test thermal stability, α-IM and IM-aerogel samples were both stored at 125 °C for up to 96 h in an oven. These samples were then removed from the oven and stored in a dessicator under vacuum before testing.

To test against seeding, we chose to introduce an amount of γ-IM crystals equal to the total weight of indomethacin in the sample to which they were added. For example, we mixed 30 mg α-IM powder with 30 mg of γ-IM under vacuum. Additionally, we mixed IM-aerogel (containing 30 mg total IM content) with 30 mg γ-IM. For α-IM, the γ-IM seeds were placed in the same container and came in contact with the α-IM. They were mixed by gently moving the powder around in the container. For the aerogel, γ-IM seeds were added directly onto the IM-aerogel sample and the aerogel was broken up and then gently mixed together with the γ-IM powder with a mortar & pestle.

DSC was performed using a Mettler Toledo DSC 3+ to determine thermal properties of the different IM samples, including melting temperature and phase transformations. The samples were heated at a rate of 3 °C/min. from 25–210 °C and as-received material was also tested for reference.

Raman spectra were collected using a Renishaw Qontor Confocal Raman Spectrometer (Wotton-under-Edge, UK) with a 785 nm laser and a grating of 1200 lines/mm. A 10 s exposure time was used with the laser operating at 50% power. The spectra were collected between 1600–1750 cm^−1^. Atef et al. have recently shown the usefulness of Raman spectroscopy in distinguishing between the α and γ form of IM. By comparing the weight % of α-IM in binary mixtures of α and γ IM to specific peak heights in a Raman spectrum, they were able to determine the signature peaks for each form (1649 cm^−1^ for α-IM and 1699 cm^−1^ for γ-IM) [7]. This method was used to quantitatively determine percentage of α-IM and γ-IM in our samples. A standard curve was first obtained by preparing binary mixtures of IM-α and IM-γ in distinct ratios (5:95, 25:75, 50:50, 70:30, 90:10) and measuring the ratio of the absolute intensity of the peaks attributed to each form immediately after mixing (1649 cm^−1^ for IM-α and 1699 cm^−1^ for IM-γ). This curve was then used to quantify the amount of each polymorphic form in our samples. Five measurements of samples from the same batch were taken for each different mixture and drug, and the average and standard deviation were reported.

Optical micrographs of the aerogels containing IM were taken using a LEICA DMi8 microscope in transmission mode (Wetzlar, Germany).

The SEM images were taken using a Zeiss Ultra60 field emission scanning electron microscope (Jena, Germany) at an operating voltage of 3 kV. The samples were mounted with carbon tape on aluminum stubs and sputter coated with a Hummer 6 gold/palladium sputter coater prior to imaging.

Dissolution studies were performed following the USP paddle method for indomethacin [12,15,55]. An Agilent 708-DS Dissolution Apparatus (Santa Clara, CA, USA) was used with a paddle attachment rotating at 100 RPM. DI water with a pH of 6.5 at 37 °C was used as the dissolution medium to simulate intestinal fluid. The aliquots were collected at various time intervals between 30 s and 2 h and an equal amount of DI water was returned to the dissolution vessel. The aliquot samples were filtered using 0.22 µm PVDF filters and measured using an Agilent Technologies Cary-60 UV-Vis spectrometer (Santa Clara, CA, USA). The measurements were taken at 318 nm. Prior to measurements, a standard curve was prepared to determine the concentration to absorbance ratio for dissolved IM. First a stock solution was prepared by dissolving 30 mg in 900 mL of the dissolution media (DI water pH 6.5 at 37 °C). Serial dilutions were made to obtain samples that ranged in concentration from 0.065–33.3 µg/mL. The samples of γ-IM were mixed with a CNC aerogel using a mortar and pestle in the same ratio of CNC to IM in the IM-aerogel sample. The concentration of dissolved drug was normalized against the original amount of drug in the sample to obtain a percentage dissolved, which has been plotted against time.

## 3. Results

### 3.1. Crystallization of α-IM within Cellulose Nanocrystal Aerogels

**Figure 1:**
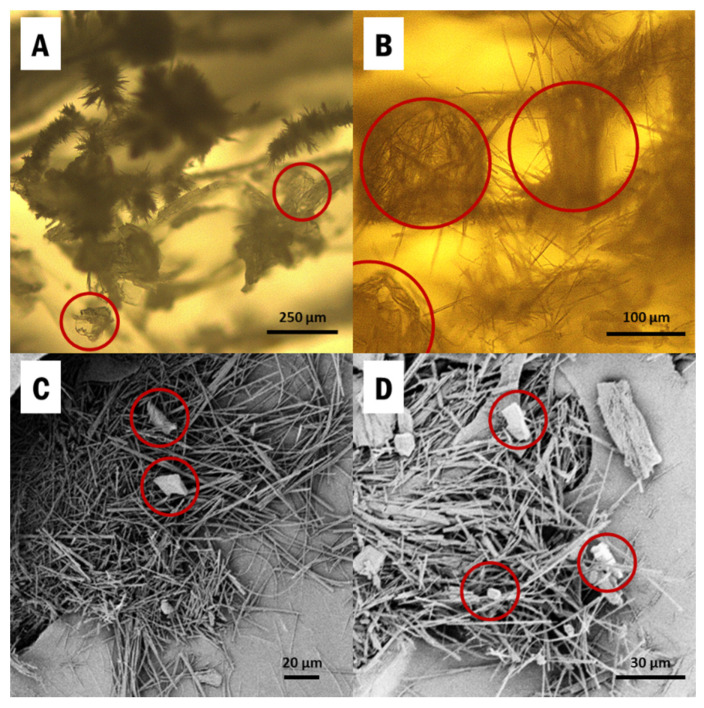
Images of Indomethacin recrystallized within cellulose nanocrystals (CNC) aerogels from: (**A**,**B**) optical microscopy; (**C**,**D**) SEM. α-Indomethacin (α-IM) crystals grow as the easily visible thin needles and γ-IM crystals are more plate-like and have been circled in red.

IM was directly drop-casted from a saturated ethanol solution into CNC aerogels, held within the silicone mold (SI Figure S1), and the samples were cooled to induce supersaturation. α-IM and γ-IM both crystallized into the aerogels and they can be seen in Figure 1. The thin needles are α-IM, which is known to have a fiber-like morphology due to very fast growth in one direction [38,56]. These are clearly visible in the SEM images (Figure 1C,D) and appear as small fuzzy spikes in the optical micrographs (Figure 1A,B). γ-IM crystals are more plate-like and easily distinguishable from the α-form, and they have been circled in red in Figure 1 [56].

**Figure 2:**
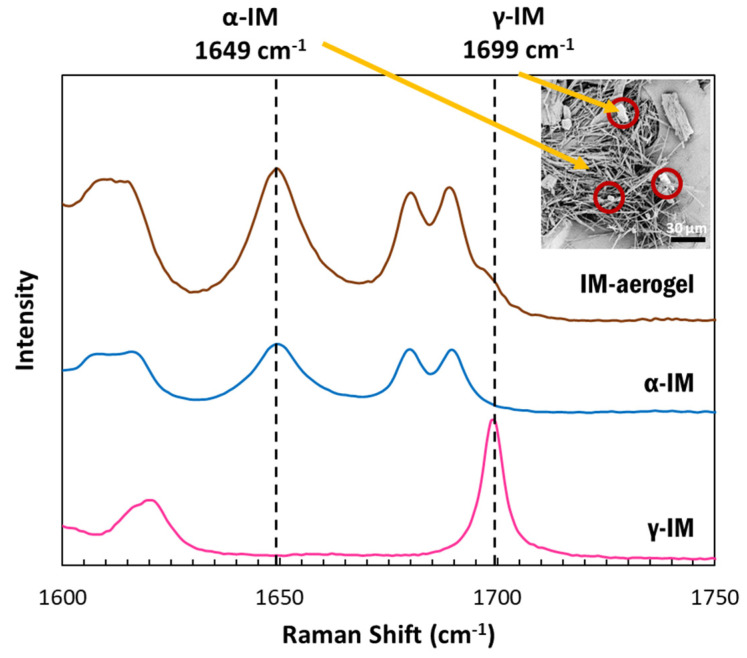
Raman spectra of IM samples between 1500-1800 cm^−1^: pink–as received γ-IM crystals (with distinct benzoyl C=O vibration peak at 1699 cm^−1^), blue–α-IM recrystallized from water (with distinct hydrogen bonded acid C=O peak at 1649 cm^−1^), and brown-IM in aerogel showing both α and γ crystal forms; inset image shows α-IM needles and γ-IM plate-crystals (red circles) within CNC aerogel.

Raman spectroscopy was used to confirm the presence of the polymorphic forms of IM using the peak at 1649 cm^−1^ to indicate α-IM and the peak at 1699 cm^−1^ to indicate γ-IM [7]. Figure 2 depicts the Raman spectra for our samples. The as-received IM (pink) showed a sharp peak at 1699 cm^−1^, which was attributed to the benzoyl C=O vibration, confirming it was the γ-form. The α-IM recrystallized from water (blue) showed no peak at 1699 cm^−1^ but a sharp peak at 1649 cm^−1^, which is attributed to the hydrogen bonded acid C=O stretch confirming this was the α-form. The IM recrystallized within the aerogel (brown) contained peaks at both 1649 cm^−1^ and 1699 cm^−1^, indicating that both α and γ forms were present within the aerogel. A Raman spectrum of the CNC aerogel is included in the SI (SI-Figure S2) and it presented no overlaps with peaks in the 1600–1750 cm^−1^ region.

**Figure 3:**
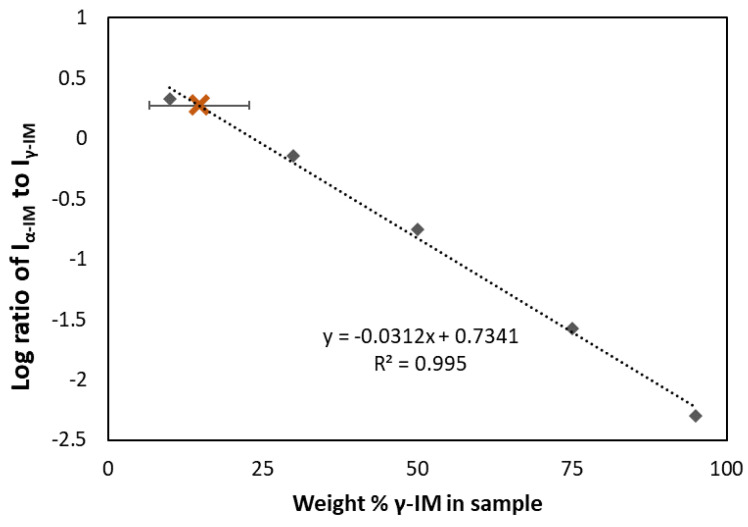
Standard curve (dashed line) showing ratio of α to γ peak intensities compared to weight percent of gamma in the binary mixture. Amount of γ-form in IM-aerogel shown using brown X and the error bar indicates standard deviation based on 5 measurements.

Raman spectroscopy was also used to quantify the amount of each form within the aerogel. A standard curve (the dashed line in Figure 3) was prepared using binary mixtures of α and γ polymorphs and plotting the ratio of the peak intensities at 1649 cm^−1^ and 1699 cm^−1^ for each sample. Using this curve, the amount of γ-IM in the aerogel was determined to be approximately 15 wt% ± 8 wt% (as indicated by the brown X in Figure 3), which indicated that most of the IM in the aerogel recrystallized as the α-form.

**Figure 4:**
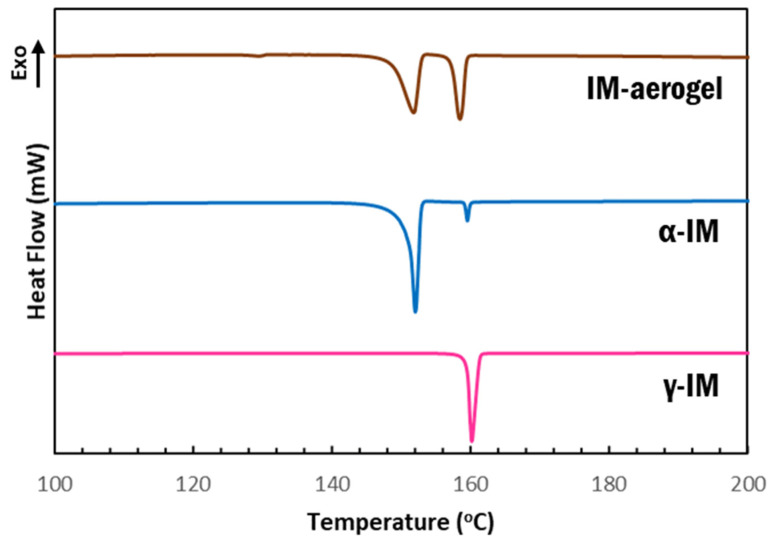
DSC thermograms of IM samples: pink-as received γ-IM; blue-recrystallized α-IM; and brown-IM recrystallized within aerogel.

DSC scans further confirmed our findings that the as received material was γ-IM with a melting temperature of 158 °C, which is the onset of the sharp endotherm in the pink curve shown in Figure 4. α-IM was obtained by using anti-solvent crystallization with H_2_O out of a saturated IM in ethanol solution. The DSC scan of this sample (Figure 4-blue) showed a melting endotherm with an onset at 147 °C, which has been documented as the α-IM melting temperature in literature [7,8,10]. A small peak with an onset temperature of 159 °C was also observed in this scan, indicating the presence of a small amount of γ-IM in the sample. This is attributed to the interconversion of α-IM to the γ-form due to heating and suggests thermal instability of the of the α-form, since the room temperature Raman spectrum of this sample (Figure 2-blue) showed no presence of γ-IM. The aerogel again contained evidence of both IM polymorphs, as seen from the two endotherms in the brown curve above. The peak with onset at 149 °C is attributed to the α form and the peak with onset at 158 °C is attributed to the γ form. A DSC thermogram of the CNC aerogel has been included in the SI (SI-Figure S3) and it shows no peaks in the 100–200 °C region that could be obscuring the data from IM.

**Figure 5:**
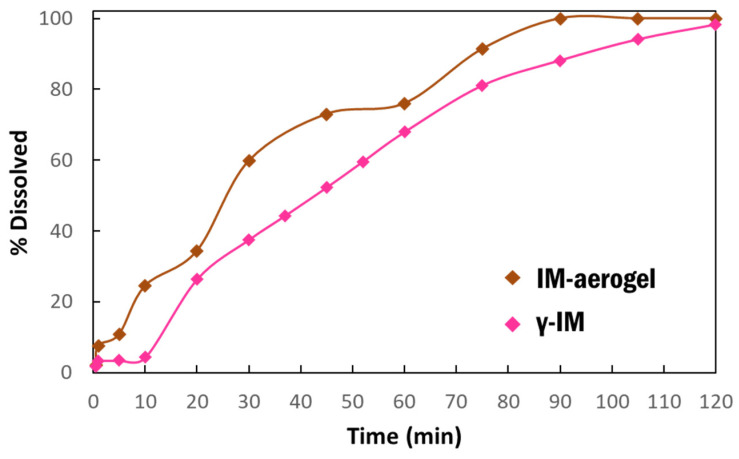
Dissolution profiles of γ-IM mixed with CNC aerogel as excipient (pink) and IM-aerogel sample (brown) in DI water at 37 °C.

Dissolution studies were performed to compare the dissolution behavior of IM in the aerogel to the as-received material (Figure 5). The dissolution was run for 2 h and it showed that the IM-aerogel samples have a faster dissolution rate, reaching 100% dissolution in 90 min. In comparison, the γ-IM samples were less than 90% dissolved at 90 min. and they reached 98% dissolution at 120 min.

### 3.2. α-IM Stabilized in CNC Aerogels at High Temperature Thermal Holds

**Figure 6:**
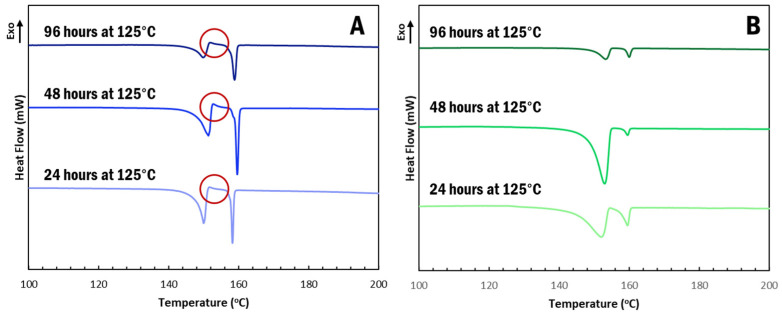
DSC scans after thermally holding (**A**). α-IM and (**B**). IM-aerogel samples at 125 °C for up to 96 H. Red circles indicate the recrystallization exotherms present in the α-IM samples.

α-IM undergoes interconversion at elevated temperatures and, so, we further studied the effect of the CNC aerogel on this transformation using thermal holds, as seen in Figure 4. Samples of α-IM powder and IM-aerogel were held at 125 °C for up to 96 h and DSC scans were performed to study any thermal effects on the materials. Interconversion was observed in all of the α-IM powder samples (Figure 6A), as evidenced by the exotherm between 152–153 °C, indicated by the red circles. This exotherm is attributed to the recrystallization of α-IM and it does not appear in the IM-aerogel samples (Figure 6B), even after 96 h at 125 °C. This indicates that the α-IM crystals within the aerogel remain in the α-form, potentially due to stabilization by the CNC aerogel.

### 3.3. α-IM Stabilized in CNC Aerogels in the Presence of γ-IM Seed Crystals

As seen in the micrographs in Figure 1, α and γ forms of indomethacin both crystallized within the aerogel; however, the stability of the metastable form needs to be further understood. Impurity crystals have been shown to cause interconversion of metastable drug forms, making them difficult to attain during production and less effective. The first part of this study showed that both α and γ forms can coexist within the CNC aerogels without interconversion, but the extent of this stability is unknown. γ-IM crystals can act as impurities or triggers for interconversion and, thus, we investigate the stability of α-IM within the CNC aerogel in the presence of γ-IM seed crystals.

**Figure 7:**
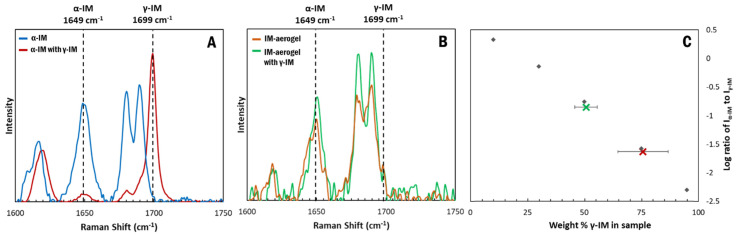
Raman spectra of IM samples stored with γ-IM seeds for 24 h: (**A**) α-IM, (**B**) IM-aerogel, and (**C**) Weight percent of γ-form in the α-IM sample (red X) and IM-aerogel (green X) calculated using the standard curve. An average weight percent was obtained using measurements from different parts of the mixture. The error bars in (**C**) are derived from the standard deviation of five measurements.

Raman spectroscopy was used to measure the transformation of the α-IM with time. When stored in the presence of γ-IM seed crystals, a bulk of the α-IM powder transitioned to the γ form within one day. This is evident in the Raman spectra of the samples (Figure 7A). Initially no γ-IM peak was observed in the α-IM sample, but, upon storage with γ-IM seeds, a sharp peak appeared at 1699 cm^−1^. Using the standard curve, the amount of γ-IM in the sample after one day was determined to be 75 wt% ± 11 wt% (Figure 7C–red X). A maximum of 50% γ-IM can be attributed to the added γ-IM seeds, since they are in present in the sample being tested; however, the excess 25 % is due to the phase transformation occurring within the material.

In contrast, when the IM-aerogel sample was stored with γ-IM seed crystals, little change in the Raman peaks was observed (Figure 7B). Because individual crystals could be monitored using the Raman microscope, this spectrum was obtained for α-IM crystals in the sample. The α-IM signature peak at 1649 cm^−1^ remains stronger than the γ-IM peak at 1699 cm^−1^. An average of the whole mixed sample was used to determine the % of γ-IM and it was found to be 51% ± 4%. This indicates that the added seed crystals are the only γ-IM in the aerogel mixture (Figure 7C–green X), in contrast to the α-IM powder, where 50% of the α-IM transformed to γ-IM during the same one day of storage with γ-IM crystals.

**Figure 8:**
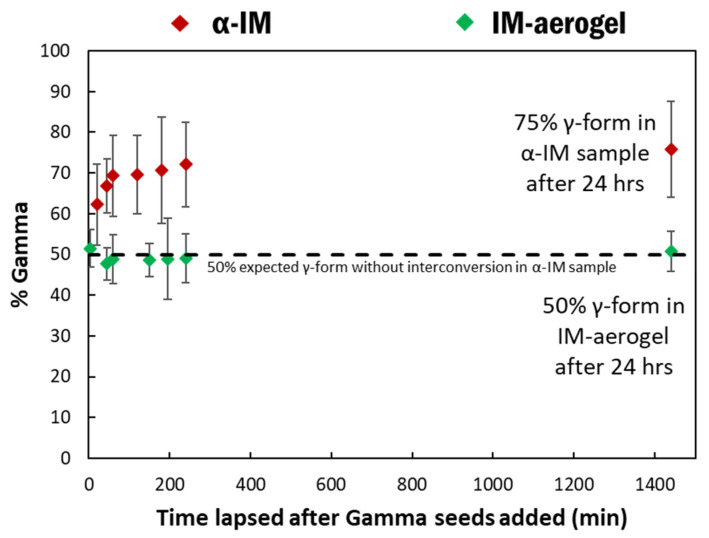
% γ-IM in the samples as a function of time after adding γ-IM seed crystals to α-IM (red) and IM-aerogel (green) samples. The black line represents the 50% mark, which would be the maximum γ-IM amount from adding the seed crystals.

The effect of shorter time frames on α-IM transformation was also studied using Raman spectroscopy. Raman spectra were taken at specific time intervals after adding the γ-IM seeds to both the α-IM and the IM-aerogel samples and the percentages of γ-IM as a function of time are shown in Figure 8. The α-IM almost immediately shows 60% γ-form in the sample. As noted previously, 50% of this could be attributed to the added γ-IM seeds, since we added an equal mass of the γ-form; however, the additional 10% was due to the interconversion effects. Over the next three hours, additional interconversion takes place, and the amount of γ-IM in the sample increases to 75% at 24 h. The IM-aerogel sample shows an average of 50% γ-form for up to 24 h. even after seeding, indicating that only the added γ-IM seed crystals are present, and no interconversion is taking place in this sample.

These results show that the metastable α-IM powder is not stable in the presence of γ-IM seeds and it undergoes interconversion to the more thermodynamically stable γ polymorph. The α-IM in the CNC aerogels, on the other hand, does not show significant transformation and it remains stabilized within the aerogel structure at room temperature, even in the presence of γ-IM seed crystals.

## 4. Discussion

**Figure 9:**
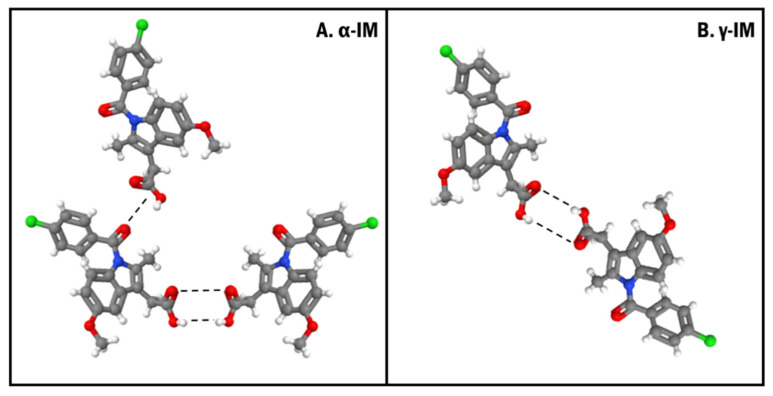
Crystal orientations showing the hydrogen bonding (dashed lines) between (**A**). α-form and (**B**). γ-form of indomethacin. Both forms contain a strongly bonded dimer between two carboxylic acid groups and the α-form also contains an additional hydrogen bond between a carboxyl group and an adjacent amide carbonyl group.

Based on the microscopy images, the crystallization of both α-IM and γ-IM was confirmed within the CNC aerogels. Raman spectroscopy was used to further evaluate these aerogel systems, and it was confirmed that the majority of the IM was α-form within the aerogel with only 15% ± 8% being γ-form. The nucleation of the metastable α-form could be promoted in part due to the inherent moisture content within CNCs. The moisture in CNCs can force precipitation of the α-form within the aerogel, as water is an anti-solvent for α-IM [57]. This metastable polymorph formation could also be due to the formation of hydrogen bonds of α-IM with the hydroxyl groups on the CNC surfaces (Figure 9). The γ-form of IM contains a strongly bonded carboxylic acid dimer within the crystal lattice. The α-form contains a hydrogen bonded trimer, where one of the carboxylic acid groups is bonded to an adjacent amide carbonyl group. Several studies have looked at the molecular conformations of these two polymorphs of indomethacin and concluded that α-IM is more reactive. This is due to a combination of accessibility of carboxyl groups within the lattice, bond lengths between the dimers and trimers, and the number of different possible conformations of the two different forms [14,57,58,59]. The carboxyl group that hydrogen bonds to the amide group in the trimer is more accessible for hydrogen bonding with the hydroxyl group on the CNC surface than carboxyl groups participating in the hydrogen bonded dimer. The crystallization of α-IM from cellulose nanofiber surfaces has been previously reported by Gao et al. They also suggested that the hydrogen bonding between the IM carboxyl group and the cellulose hydroxyl group was the primary driving force for this interaction [60].

While the hydrogen bonding between CNC and α-IM is crucial to the crystallization of the metastable phase, the dissolution studies showed that IM-aerogel is able to achieve faster dissolution than the γ-form. CNCs are dispersible in water and the CNC aerogel networks can be easily disrupted by water. Therefore, the α-IM crystals from within the aerogel can be accessed and dissolved once the CNC aerogel is broken down in the simulated intestinal fluid. This network disruption also breaks the hydrogen bonding with the α-IM, and it does not seem to affect the crystal’s ability to dissolve.

Our current investigation also showed the stability of α-IM within the aerogels in contrast to α-IM powder that underwent interconversion to the γ-form when stored under heat or in the presence of γ-IM seed crystals, which are common scenarios that arise during the production and processing of indomethacin. The α-form of IM is more soluble, and access to this form can provide a higher efficacy drug formulation, but the stability is an issue during actual production. As a result, the practical application of α-IM is limited since natural processing conditions could lead to a transformation of the metastable form. While Ostwald’s rule and kinetics favors the nucleation of α-IM since it is the lower stability form, when storing this material at elevated temperatures, thermodynamics dictates that the most stable γ-form of IM would result [8,10,16]. This is observed in the DSC scans presented in Figure 6, where thermal holds at 125 °C result in additional interconversion of α-IM to γ-IM. Seeding also causes this interconversion, and timed studies showed that, in the presence of γ-IM seed crystals, some α-IM crystals transition to a different crystal structure almost immediately and, after 24 h, 50% of the α-IM crystals have undergone interconversion, resulting in a total of 75% γ-IM in the sample.

The formation of hydrogen bonds between CNCs and the carboxyl groups on α-IM would also explain the stability against this transformation. Interconversion within the aerogels would involve the disruption of these hydrogen bonds, requiring additional energy. As such, even though γ-IM is the more thermodynamically stable form, the α-IM does not undergo interconversion and remains bonded to the CNC surfaces on the aerogel. This hydrogen bonding is also able to provide stability against the seed-induced interconversion. α-IM and γ-IM are both present within the aerogel to begin with, which indicates that these α-IM crystals are stable and do not change to the γ-form. Furthermore, even when additional γ-IM seed crystals are added, the α-form remains stabilized within the aerogel. Seeding typically provides a platform for growth and it can cause a shift in the crystal structure; however, no such shift occurs since the α-IM crystals are already physically bound to the CNCs.

Additionally, the porous structure of the aerogel can further assist in stabilizing the indomethacin crystals. Because the α-IM crystals exist as long fiber-like needles, the constraints of the pore walls within the CNC aerogel would limit the mobility of IM crystals and, therefore, restrict interconversion to more the plate-like γ-IM crystals. This type of stabilization within pores has previously been suggested by Nartowski et al. for IM form V [27]. They posited that the spatial constraints inside controlled glass pores assisted with crystallizing IM form V and prevented impurities from affecting the crystals. Because the CNC aerogels can also provide spatial constrains to the α-IM needles, it is possible that these crystals are further prevented from conversion.

## 5. Conclusions

These results show the effects of the CNC aerogels on stabilizing the metastable α-form of the model drug indomethacin. IM was recrystallized into CNC aerogel scaffolds as a mixture of the two polymorphic forms α-IM and γ-IM. It was shown that α-IM powder has a tendency to interconvert to the more stable γ-form when stored with γ-IM seed crystals. Through the use of quantitative Raman spectroscopy analysis, we showed that, when the α-IM is crystallized in a CNC aerogel, no interconversion occurs in the presence of γ-IM seeds. This study also investigated the effect of the CNC aerogel on stabilizing α-IM during high temperature holds in order to further stress the material and determine whether this stabilization effect was effective with under elevated temperature conditions. While α-IM powder by itself showed high interconversion and recrystallization to the γ-form, when held at 125 °C for up to 96 h, the α-form within CNC aerogels did not show any such transformation. This has important implications not only in being able to stabilize a metastable drug form, but also in increasing the efficacy of certain APIs, as these metastable forms, typically with higher solubilities, could be administered to patients without the concern of interconversion. The ability to use these CNC aerogels to protect against interconversion due to the presence of seeds of a more stable API form is an added benefit, especially during the production process.

## Figures and Tables

**Table 1:** Crystal data and physical properties of the α and γ polymorphs of indomethacin [8,9,12,14,15,16].

IM Polymorphic Form	Melting Temperature	Space Group	Unit Cell Dimensions	Z, Z’	Solubility in Water at 25 °C
α	152–154 °C	P2_1_	a = 5.4616 ± 0.0016 Å b = 25.31 ± 0.009 Å c = 18.152 ± 0.007 Å α = 90° β = 94.38 ± 0.03° γ = 90°	6, 3	0.8 ± 0.01 mg/mL
γ	160–161 °C	P$\bar{1}$	a = 0.295 ± 0.002 Å b = 10.969 ± 0.001 Å c = 9.742 ± 0.001 Å α = 69.38 ± 0.01° β = 110.79 ± 0.01° γ = 92.78 ± 0.01°	2, 1	0.4 mg/mL

## Data Availability

The data presented in this study are available within the article and the supplementary information.

## Supplementary Materials

The following are available online at https://www.mdpi.com/1999-4923/13/4/441/s1, Figure S1: Images of cellulose nanocrystal aerogels a. In silicone tray used for casting, b. Side view, c. top down view, Figure S2: Raman spectra of cellulose nanocrystal aerogel showing no peaks in the region of interest for indomethacin polymorphs (1600–1750 cm^−1^), Figure S3: DSC thermogram of CNC aerogel showing no peaks in region of interest for indomethacin polymorphs (140–162 °C).
