# Supplementary material for: Stabilization of Metastable Indomethacin α in Cellulose Nanocrystal Aerogel Scaffolds
Source: Pharmaceutics. 2021 Mar 24;13(4):441. doi: 10.3390/pharmaceutics13040441 (PMC8064329; doi:10.3390/pharmaceutics13040441)
Supplement: Supplementary file 1 [file pharmaceutics-13-00441-s001.pdf]

pharmaceutics

MDPI

Supplementary Materials: Stabilization of Metastable Indomethacin α in Cellulose Nanocrystal Aerogel Scaffolds

Manali Banerjee and Blair Brettmann

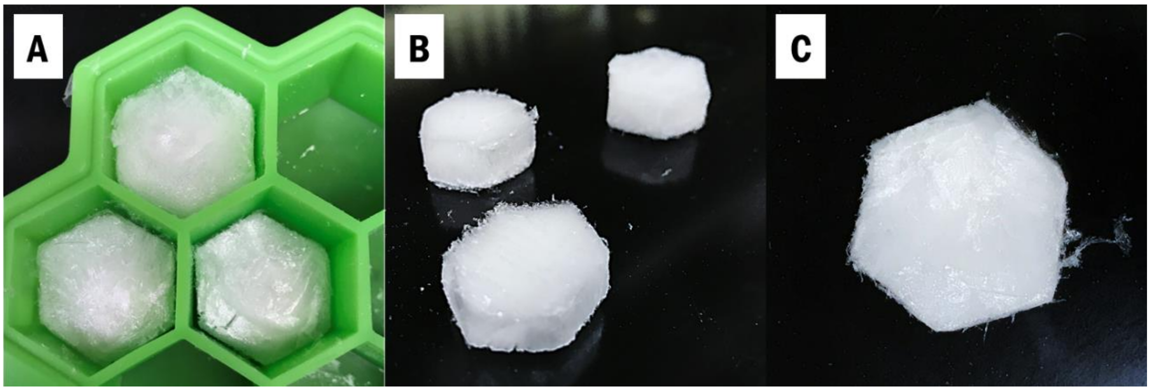

Figure S1. Images of cellulose nanocrystal aerogels a. In silicone tray used for casting, b. Side view, c. top down view

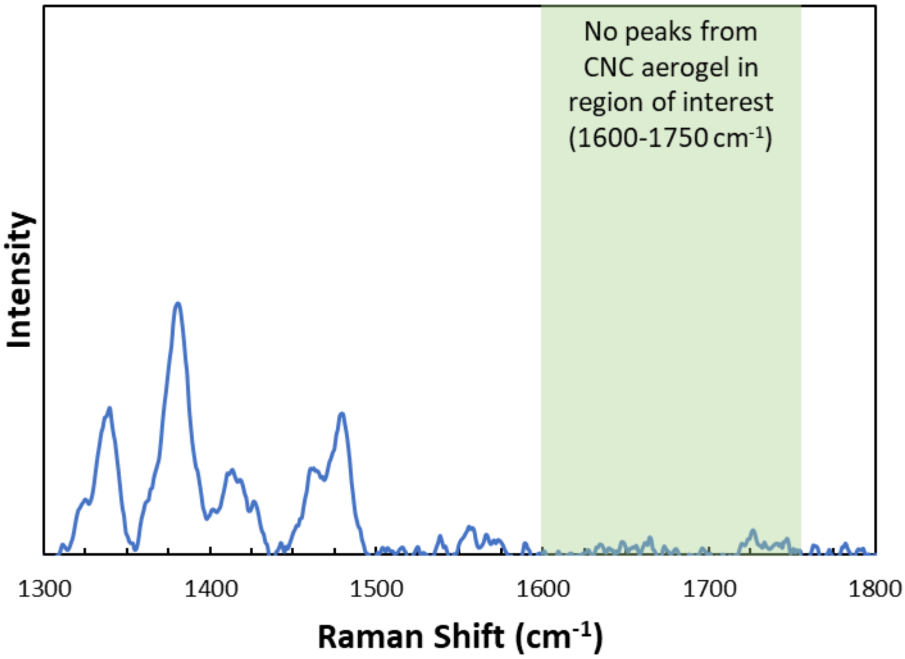

Figure S2. Raman spectra of cellulose nanocrystal aerogel showing no peaks in the region of interest for indomethacin polymorphs (1600–1750 cm^{-1})

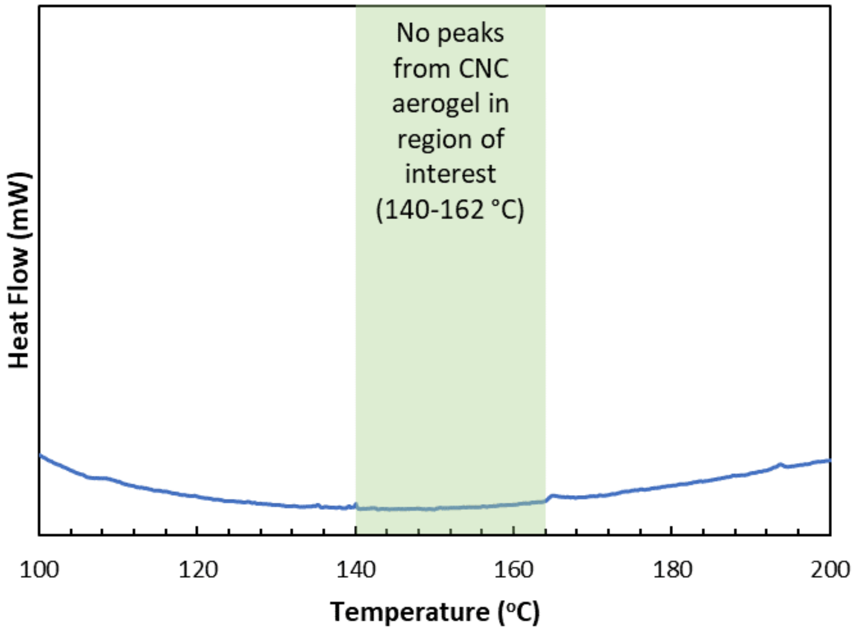

Figure S3. DSC thermogram of CNC aerogel showing no peaks in region of interest for indomethacin polymorphs (140–162 °C)
